# GPR35‐mediated kynurenic acid sensing contributes to maintenance of gut microbiota homeostasis in ulcerative colitis

**DOI:** 10.1002/2211-5463.13673

**Published:** 2023-07-14

**Authors:** Di Wang, Wenbao Wang, Xue Bing, Chenguang Xu, Jiahua Qiu, Jiangang Shen, Jinwen Huang, Junda Li, Pi Liu, Biao Xie

**Affiliations:** ^1^ School of Life and Health Technology Dongguan University of Technology Dongguan China; ^2^ Department of Gastroenterology Affiliated Longhua People's Hospital, Southern Medical University (People's Hospital of Longhua) Shenzhen China; ^3^ College of Pharmacy Qiqihar Medical University Qiqihaer China; ^4^ Department of Gastroenterology Guangzhou Eighth People's Hospital, Guangzhou Medical University Guangzhou China

**Keywords:** GPR35, gut homeostasis, gut microbiota, kynurenic acid, ulcerative colitis

## Abstract

Ulcerative colitis (UC) is a recurrent inflammatory disease related to gut microbiota disorder. Metabolites and their sensors play an important role in the communication between gut microbes and their host. Our previous study revealed that G protein‐coupled receptor 35 (GPR35) is a key guardian of kynurenic acid (KA) and a core element of the defense responses against gut damage. However, the mechanism remains unknown. In this study, a DSS‐induced rat colitis model was established and 16S rRNA sequencing was applied to explore the influence of GPR35‐mediated KA sensing on gut microbiota homeostasis. Our results demonstrated that GPR35‐mediated KA sensing is a necessary component in maintaining gut barrier integrity against DSS‐induced damage. Furthermore, we provide compelling evidence suggesting that GPR35‐mediated KA sensing plays a crucial role in maintaining gut microbiota homeostasis, which contributes to alleviation of DSS‐induced colitis. In addition, five classes (Actinobacteria, Beta‐/Gamma‐proteobacteria, Erysipelotrichi, and Coriobacteriia) and six genera (*Corynebacterium*, *Allobaculum*, *Parabacteroides*, *Sutterella*, *Shigella*, and *Xenorhabdus*) were identified as the marked bacterial taxa that characterized the progression and outcome of colitis and are regulated by GPR35‐mediated KA sensing. Our findings highlight that GPR35‐mediated KA sensing is an essential defense mechanism against disorder of gut microbiota in UC. The results provide insights into the key role of specific metabolites and their monitor in maintaining gut homeostasis.

## Graphical abstract

Metabolites and their sensors play an important role in the communication between gut microbes and their host. GPR35‐mediated KA sensing plays a crucial role in maintaining gut microbiota homeostasis, which contributes to alleviation of DSS‐induced colitis. Besides, five classes and six genera were identified as the marked bacterial taxa that characterized the progression and outcome of colitis and are regulated by KA and its sensor GPR35.

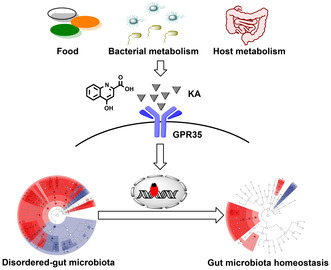

**Abbreviations**
AB: Alcian blue staining
CID: CID2745687
DAI: disease activity index
DSS: dextran sulfate sodium
GPR35: G protein‐coupled receptor35
GWAS: Genome‐wide association studies
H&E: Hematoxylin–eosin staining
IBD: inflammatory bowel disease
KA: kynurenic acid
KYN: kynurenine
LDA: linear discriminant analysis
LEfSe: linear discriminant analysis effect size
NMDS: non‐metric multidimensional scaling
PCoA: principal coordinate analysis
PICRUSt2: phylogenetic investigation of communities by reconstruction of non‐observed states
Trp: tryptophan
UC: ulcerative colitis

Ulcerative colitis (UC) is a persistent and recurrent inflammatory disease, which has been classified as one of the refractory diseases by the World Health Organization [1]. Its main manifestations are abdominal pain, diarrhea, mucosanguineous feces, and weight loss. In recent years, the number of UC patients has increased rapidly, and this disease is becoming one of the most challenging diseases in the world. However, the exact pathogenesis of UC remains poorly understood. Epidemiological studies have shown that immune dysfunction, genetic susceptibility, and microbial imbalance may be responsible for the occurrence of UC [2]. Accumulating evidence supports the hypothesis that UC is caused by persistent and inappropriate immune activation through a weakened intestinal barrier based on the crosstalk of the host and the gut microbiota, which can be affected by environmental factors and genetic susceptibility [3]. In addition, the dysregulation of microbiota composition and diversity is closely associated with the increase of intestinal permeability and the activation of the inflammatory response [4]. Therefore, maintaining gut function and barrier integrity are crucial for host defense and preserving microbiota homeostasis.

Metabolites and their sensors play a crucial and irreplaceable role in communication between intestinal microbe and the host [5, 6]. The disorders in the intestinal microbiota caused by the majority of factors can be transmitted to the host through metabolites and their monitors. This, in turn, drives a feedback response that resists the stimulus and redress the abnormities [7, 8]. Among the various of metabolic sensors, G protein‐coupled receptors (GPCRs) have achieved significant attention for their unique ability to establish communication between the host and its environment [5, 9]. G protein‐coupled receptor 35 (GPR35) has shown significantly high expression in the gastrointestinal tract with distinct tissue specificity. Genome‐wide association studies have identified GPR35 single nucleotide polymorphisms as a risk factor for inflammatory bowel disease (IBD), suggesting that GPR35 plays an important role in intestinal homeostasis [10]. Previous studies have shown that GPR35 contributed to gut mucosal repair in mice [11]. GPR35 deficiency aggravated dextran sulfate sodium (DSS)‐induced colitis and led to neutrophil recruitment, as well as significantly decreased efficiency in clearing the number of peritoneal bacteria [12, 13]. Additionally, Melhem *et al*. [14] indicated that the presence of GPR35 in epithelial cells was a critical element in the function of goblet cell‐mediated symbiosis between the host and the microbiota. However, the interactions between the gut microbiota, intestinal barrier, and host GPR35 remain incompletely understood.

Kynurenic acid (KA), a product of tryptophan catabolism via kynurenine pathway, is an endogenous ligand of GPR35. Both the host and gut‐resident bacteria, such as *Escherichia coli*, are the main manufacturing sources of KA [15]. KA is widely distributed in food, including high levels reported in honey, broccoli, and potato [16]. Moreover, dandelion, nettle, greater celandine, and other Chinese herbs that possess certain therapeutic effects against specific gastrointestinal diseases, including UC, have also been noted to contain high levels of KA [17]. Pharmacological studies have shown that KA has a positive impact on multiple gastrointestinal pathologies, particularly ulcers, colon obstruction, and colitis [18]. For instance, Glavin *et al*. reported that KA protected against gastric and duodenal ulceration caused by a poisonous Atlantic shellfish and attenuated the formation of stress‐ and ethanol‐induced ulcers in rats [19, 20]. Therefore, the presence of KA is beneficial for sustaining gut health. However, the mechanism and effect of KA on gut homeostasis remain ill‐defined. Recently, studies have proposed that tryptophan and its metabolites, including KA, play an essential role in intestinal homeostasis, partly via activating GPR35 [21]. Our previous study has demonstrated that following the development of UC, the level of KA was significantly increased in the colon and that GPR35 could drive a defensive response to resist gut damage by sensing KA level, specifically [22]. However, regrettably, the potential mechanism of GPR35‐mediated KA sensing in regulating intestinal homeostasis remains unclear.

Therefore, the current study utilized the DSS‐induced rat colitis model to assess the influence of GPR35‐mediated KA sensing on gut microbiota homeostasis following KA administration and inhibition of the host GPR35 activity. Our study demonstrated that GPR35‐mediated KA sensing was a necessary component in maintaining gut barrier integrity, and an essential defender against gut microbiota disorders in UC. Our findings highlight the significance of metabolites and their sensors in maintaining gut homeostasis.

## Materials and methods

### Animals

All animal studies and procedures were conducted in accordance with the ARRIVE guidelines and the US National Institutes of Health Guide for the Care and Use of Laboratory Animals and approved by the Ethics Committee of the People's Hospital of Longhua, Shenzhen (License No.: LHRY‐2102010). Male Sprague–Dawley (SD) rats at the age of 6–8 weeks were purchased from Guangdong Province medicine experimental animal center (permission No.: SCXK (Yue) 2018–0002). All rats were housed in a temperature‐controlled environment (room temperature of 23 ± 2 °C; relative humidity of 50 ± 5%). The animals were allowed a week to acclimatize to a standard rodent diet under a 12 h/12 h dark/light cycle prior to initiation of the experiments.

### Dextran sulfate sodium‐induced rat colitis model

The rats were randomly divided into four groups as follows: Control group, DSS model group, KA treatment group and CID2745687 + KA treatment group (*n* = 12). The colitis model was established as previously reported with slight modifications [22], and the rat disposal process is shown in Fig. S1. The model and drug treatment group rats were administered with 5% (w/v) DSS (MW = 40 kDa; Aladdin, Shanghai, China) solution in drinking water for 7 consecutive days starting from the third day of the experiment, and the control group received normal drinking water. The KA treatment group was administered with KA (50 mg·kg^−1^·day^−1^, intragastrically) for 9 and16 consecutive days, and the CID2745687 + KA group was treated with CID2745687 (10 mg·kg^−1^·day^−1^, intraperitoneally) and KA (50 mg·kg^−1^·day^−1^, intragastrically) for 9 and16 consecutive days, respectively. The control group was intraperitoneally injected with the vehicle consistently. The body weight and disease activity index (DAI) were recorded daily. DAI was determined by combining the scores for the body weight loss, stool consistency, and gross bleeding according to previous reports [23]. On days 10 and 17, six rats from each group were randomly selected for further analysis of intestinal permeability. Moreover, the other rats were sacrificed and their colon tissues and colon content were collected.

### Microbiome analysis (16S rRNA sequencing)

#### DNA extraction

The genomic DNA of colonic content was extracted using the OMEGA Soil DNA Kit (Omega Bio‐Tek, Norcross, GA, USA), according to the instructions. NanoDrop NC2000 spectrophotometer (Thermo Fisher Scientific, Waltham, MA, USA) was used to analyze the concentration of the extracted DNA. Agarose gel electrophoresis was performed to examine the quality of DNA.

#### 16S rRNA gene amplicon sequencing

PCR amplification of the bacterial 16S rRNA gene V3–V4 region was performed using the forward primer 338F (5′‐ACTCCTACGGGAGGCAGCA‐3′) and the reverse primer 806R (5′‐GGACTACHVGGGTWTCTAAT‐3′). Sample‐specific 7‐bp barcodes were incorporated into the primers for multiplex sequencing. The PCR components contained 5 μL of buffer (5×), 0.25 μL of Fast pfu DNA Polymerase (Novoprotein, Suzhou, China), 2 μL of dNTPs (TOYOBO, Shanghai, China), 1 μL (10 μm) of each Forward and Reverse primer, 1 μL of DNA Template, and 14.75 μL of ddH_2_O. Thermal cycling consisted of an initial denaturation at 98 °C for 5 min, followed by 25 cycles consisting of denaturation at 98 °C for 30 s, annealing at 53 °C for 30 s, and extension at 72 °C for 45 s, with a final extension of 5 min at 72 °C. The purification and quantification of PCR amplicons were performed using VAHTSTM DNA Clean Beads (Vazyme, Nanjing, China) and Quant‐iT PicoGreen dsDNA assay kit (Invitrogen, Carlsbad, CA, USA), respectively. After the individual quantification step, amplicons were pooled in equal amounts, and pair‐end 2 × 250 bp sequencing of the amplicons were performed using the lllumina NovaSeq platform with NovaSeq 6000 SP Reagent Kit (lllumina, San Diego, CA, USA) at Shanghai Personal Biotechnology Co., Ltd (Shanghai, China).

#### Data analysis and bioinformatics

qiime2 and r packages (v3.2.0) were used to analyzed the sequencing data [24]. The alpha diversity indices including Chao1, Observed_species, Shannon, Simpson, Faith_pd, Goods_coverage and Pielou_e were calculated using the ASV table in QIIME2. Beta diversity of the microbial communities was analyzed by Bray–Curtis metrics and visualized by principal coordinate analysis (PCoA) and non‐metric multidimensional scaling (NMDS) [25]. megan and graphlan analyses were used to investigate the taxonomy compositions and abundance of gut microbiota [26, 27]. The differentially taxa in different samples were detected by LEfSe (Linear discriminant analysis effect size) analysis [28]. Random forest analysis was used to evaluate the importance of the taxa on the established model using qiime2. Based on KEGG database [29], the functions of differential microbiota across the groups were analyzed by picrust2, and the differential signaling pathways were enriched according to adj *P*‐value <0.05 and logFC (fold change) > 2.

### Intestinal permeability analysis

Rats were jejunitas for 6 h, and subsequently, FITC‐dextran (MW: 4000; Sigma, St. Louis, MO, USA) was administered by oral gavage at a concentration of 100 mg·mL^−1^ in water (750 mg·kg^−1^). Four hours later, their serum samples were collected. The fluorescence intensity was analyzed using a microplate reader (Thermo Fisher Scientific, MA, USA) at an excitation and emission wavelength of 485 nm and 535 nm, respectively. The concentration of FITC‐dextran in the serum was calculated according to a standard curve.

### Hematoxylin–eosin staining

The colon tissues were fixed in 10% buffered formalin for 3 days. Hematoxylin–eosin (H&E) staining was performed according to traditional methods [30]. Briefly, the tissues were embedded in paraffin and subsequently sectioned at a thickness of 4 μm. The sections were prepared in a specific order by dewaxing with graded xylene and ethanol, and stained with H&E solution. Following staining, they were dehydrated and mounted with neutral resin. The section tissue morphology was observed and captured by an inverted microscope (Leica, Shanghai, China).

### Alcian blue staining

Alcian blue (AB) staining was performed according to conventional methods [31]. Briefly, the colon tissues were fixed in Carnoy's fixative (Servicebio, Wuhan, China) and sectioned at a thickness of 4 μm. The sections were prepared in a specific order by dewaxing with graded xylene and ethanol and stained with Alcian blue dye solution A (Sigma, St. Louis, USA) for 10 min and solution B for 3 min, successively. Subsequently, the sections were dehydrated, and mounted with permount, and their microstructures were captured using an inverted microscope (Leica).

### Western blotting

The colon tissues were lysed by radioimmunoprecipitation buffer (Beyotime Biotechnology, Shanghai, China) containing 1 mmol·L^−1^ phenylmethylsulfonyl fluoride (Beyotime Institute of Biotechnology). After quantified by a BCA protein assay kit (Thermo Fisher Scientific, Waltham, MA, USA) and denaturalized at 100 °C for 10 min, the lysates (30 mg) were separated by SDS‐polyacrylamide gel electrophoresis (Bio‐Rad, Hercules, CA, USA) and transferred to polyvinylidene difluoride membranes (Millipore, Bedford, MA, USA) [32]. The membranes were incubated with the primary antibodies of anti‐β‐actin, anti‐ERK1/2, and anti‐p‐ERK1/2 (Cell Signaling Technology, Boston, MA, USA) at 4 °C overnight, and then subjected to horseradish peroxidase (HRP)‐conjugated secondary antibody (Proteintech, Shanghai, China) for 2 h at room temperature. An enhanced chemiluminescence (ECL) system (Millipore, Bedford, MA, USA) was used to visualize bands on a chemiluminescent imaging apparatus (AmershamImageQuant 800, Wisconsin, MA, USA). The relative protein expression was analyzed by imagej software.

### Statistical analysis

graphpad prism 9.0 software (GraphPad Software Inc., San Diego, CA, USA) was used for statistical analyses. Data were expressed as mean ± standard deviation (SD). The independent unpaired two‐tailed Student's *t*‐test was performed to evaluate the differences between the two groups. Multiple group comparisons were analyzed by one‐way analysis of variance (ANOVA) followed by the Tukey–Kramer test. *P* < 0.05 was considered to indicate a statistically significant difference.

## Results

### GPR35‐mediated KA sensing alleviates DSS‐induced colitis

Our previous study revealed that GPR35 is a key guardian of KA metabolism and of the core components involved in the defense responses against intestinal damage [22]. However, the mechanism of GPR35‐mediated KA sensing in regulating intestinal homeostasis remains unknown. With the development of biology, the irreplaceable role of gut microorganisms in maintaining intestinal function and integrity has increasingly been emphasized [33].

**Fig. 1:**
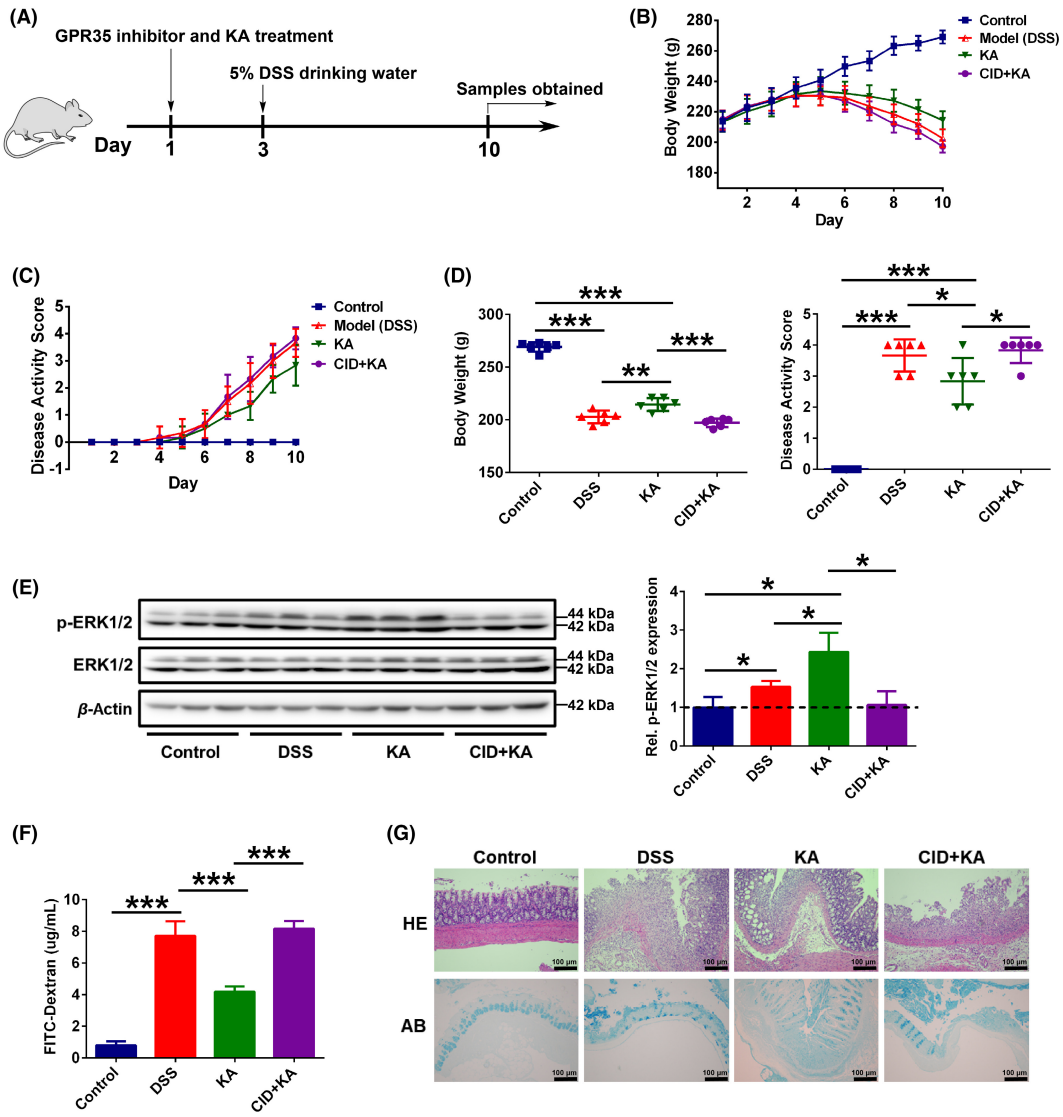
KA alleviates DSS‐induced colitis via GPR35‐mediated sensing. (A) Schematic representation of the experimental design used. (B, C) The body weight (B) and disease activity score (C) of each rat were monitored daily, CID represents the GPR35 specificity inhibitor CID2745687. (D) The body weight and disease activity score were analyzed at the end of the experiment. (E) Western blot and quantification analyses of the ERK1/2 and p‐ERK1/2 protein expressions in the rat colon. (F) Intestinal permeability analysis. (G) H&E and AB staining analyses of the rat colon tissues, scale bars = 100 μm (as indicated). The data are presented as mean ± SD. Statistical analysis was performed using the Student's *t* test. *n* = 6, *P < 0.05, **P < 0.01 and ***P < 0.001.

Therefore, in order to clarify the influence of GPR35‐mediated KA sensing on gut microbiota, we firstly established a DSS‐induced rat colitis model, and the rat administrative program is shown in Fig. 1A and Fig. S1. It was observed that the model group rats gradually became languid and emaciated, with their feces characterized by watery stool, hemorrhage, and mucus following DSS treatment. However, these symptoms were dramatically alleviated following KA administration. Daily records indicated that the body weights of the model group rats were rapidly reduced following DSS treatment, whereas their disease activity scores (DASs) were rapidly elevated. Although similar findings (body weight loss and DAS increase) were also noted in the KA treatment group, these trends were significantly alleviated (Fig. 1B,C). Moreover, at the end of the experiment, there were significant differences in body weight and DAS between the model and KA groups (*P* < 0.05), and the change in these two indicators caused by DSS was significantly alleviated under the KA administration (Fig. 1D). More importantly, the intervention of the GPR35 specific inhibitor (CID2745687, CID) dramatically suppressed KA‐induced mitigatory effect on body weight loss and DAS rising (Fig. 1B–D). These results demonstrate that GPR35 is a crucial regulatory element in the ability of KA to resist UC progression.

To further investigate the effects of GPR35‐mediated KA sensing in DSS‐induced gut damage, the activation of GPR35 in colon tissues after drugs treatment was examined. It has been confirmed that the phosphorylation of extracellular signal‐regulated kinase 1/2 (p‐ERK1/2) is one of the downstream effector molecules of GPCR and is widely used to characterize the activation of GPR35 [34, 35, 36]. The western blotting results showed that compared to the control group, the phosphorylation levels of ERK1/2 in the rat colon tissues of the model group were significantly elevated (Fig. 1E). This could be due to the effect of colitis in promoting Trp‐KYN‐KA (Tryptophan‐Kynurenine‐Kynurenic acid) axis metabolism, which led to an increase in KA levels in the host [22]. More importantly, compared to the model group, p‐ERK1/2 expression levels were significantly increased in the rat colon tissues of the KA group (Fig. 1E), indicating that KA treatment further promoted the activation of colon GPR35. Furthermore, CID intervention markedly blocked the KA‐induced GPR35 activation in the colon. Intestinal permeability analysis indicated that colonic permeability was significantly increased following the development of colitis, which was dramatically relieved after KA treatment (Fig. 1F). Inhibiting the activation of GPR35 disrupted the therapeutic effects of KA on the DSS‐induced dysfunction of colon permeability. In addition, histopathological examination indicated that KA obviously ameliorated DSS‐induced colon damage severity and goblet cell or mucous layer defectiveness (Fig. 1G). However, these effects were significantly suppressed when the rats were treated with CID. Collectively, these results indicate that GPR35‐mediated KA sensing is necessary to maintaining intestinal homeostasis against UC‐induced gut damage.

### GPR35‐mediated KA sensing improves DSS‐induced disturbance in the gut microbiota

**Fig. 2:**
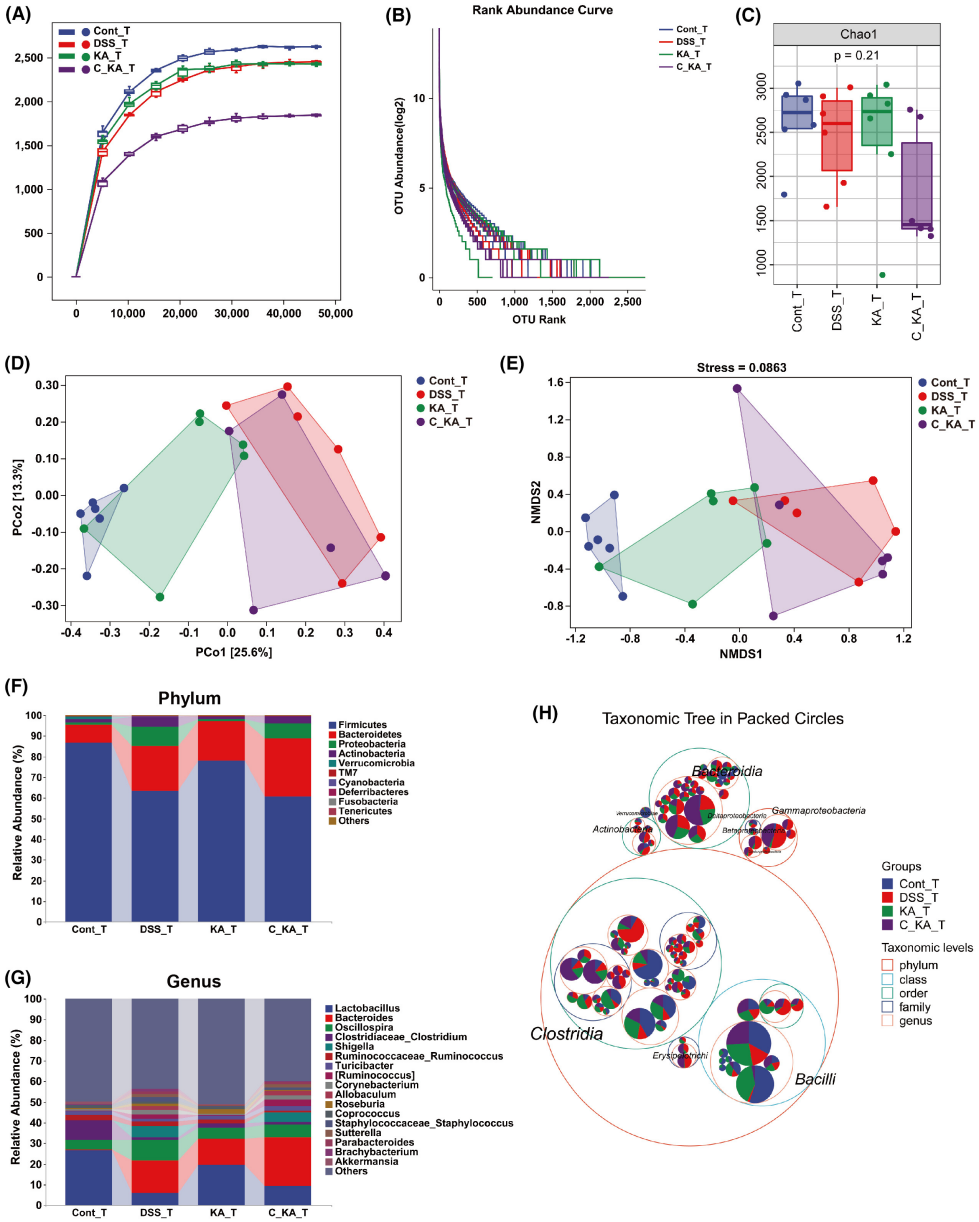
GPR35‐mediated KA sensing improves gut microbial composition in DSS‐induced colitis rats. (A, B) Rarefaction curve (A) and Rank abundance curve (B) of each group. (C) α‐diversity analysis of gut microbiota by Chao1 index. (D, E) The structural variation of the microbial communities across the samples was analyzed by PCoA (D) and NMDS (E). (F, G) The composition of gut microbiota at the phylum (F) and genus (G) levels among the different groups. (H) The distribution of the taxonomic units in the classification levels was analyzed by the taxonomic tree. The suffix of “T” in each group represents that the samples for analysis were obtained on day 10.

To investigate the impact of GPR35‐mediated KA sensing on the composition of gut microbiota in colitis rats, 16S rRNA gene sequencing was performed on colon content. The rarefaction curve was gradually flattened out with the increase of sequencing volume, indicating that the sequencing data were acceptable (Fig. 2A). Moreover, the rank abundance curve demonstrated that the sample size used for sequencing was sufficient and that the species in the sample exhibited wide coverage (Fig. 2B). The α‐diversity evaluation showed no significant differences among the four groups with regard to the 7 indices examined, such as the Chao1 index (*P* > 0.05), suggesting that GPR35‐mediated KA sensing exhibited no obvious effect on the α‐diversity of the gut microbiome during the progression of colitis (Fig. 2C, Fig. S2). However, importantly, principal coordinate analysis based on unweighted UniFrac metrics (PCoA) demonstrated that DSS led to significant separation of the gut microbiota between the control and model groups, while KA treatment notably improved this separation (Fig. 2D). In addition, the CID intervention group exhibited obvious separation with the KA treatment group, while had no significant difference compared to the model group. These results indicate that inhibiting GPR35 activation significantly blocks the meliorative effects of KA on DSS‐induced disturbance of the gut microbiota. Moreover, NMDS analysis displayed similar results to those noted by PCoA analysis, which further confirmed our conclusion (Fig. 2E).

To determine the effects of GPR35‐mediated KA sensing on colonic microbial composition and structure, the taxonomic composition was analyzed at the phylum and genus levels. As the result shown in Fig. 2F, the dominant phyla in all groups were Firmicutes, Bacteroidetes, Proteobacteria, and Actinobacteria. Compared to the control group, the proportions of Bacteroidetes, Proteobacteria, and Actinobacteria were increased, whereas that of Firmicutes was decreased in the model group. However, KA obviously suppressed the DSS‐induced increase in the abundance of Proteobacteria and Actinobacteria, and the DSS‐induced decrease in the abundance of Firmicutes. These effects were blocked when the activity of host GPR35 was inhibited. For the dominant genus, the occurrence of colitis led to a reduction in the abundance of *Lactobacillus* and *Clostridiaceae_Clostridium*, and an increase in the abundance of *Bacteroides*, *Oscillospira*, and *Shigella* (Fig. 2G). Consistently, this dysbacteriosis in colon was ameliorated by KA. Furthermore, inhibiting host GPR35 activity also reversed the regulation of KA in the gut bacterial genera. Further analysis by taxonomic tree indicated that the Beta‐/Gamma‐Proteobacteria, Actinobacteria and Erysipelotrichi classes were intensely enriched in the model and CID + KA groups (Fig. 2H), while very small amounts of these bacterial species were noted in the control and KA groups. We noticed that the model and CID + KA groups were characterized by serious UC‐associated symptoms (Fig. 1B–D). Collectively, these data support the hypothesis that the homeostasis of Beta‐/Gamma‐Proteobacteria, Actinobacteria, and Erysipelotrichi in the progression of UC is closely associated with GPR35‐mediated KA sensing.

**Fig. 3:**
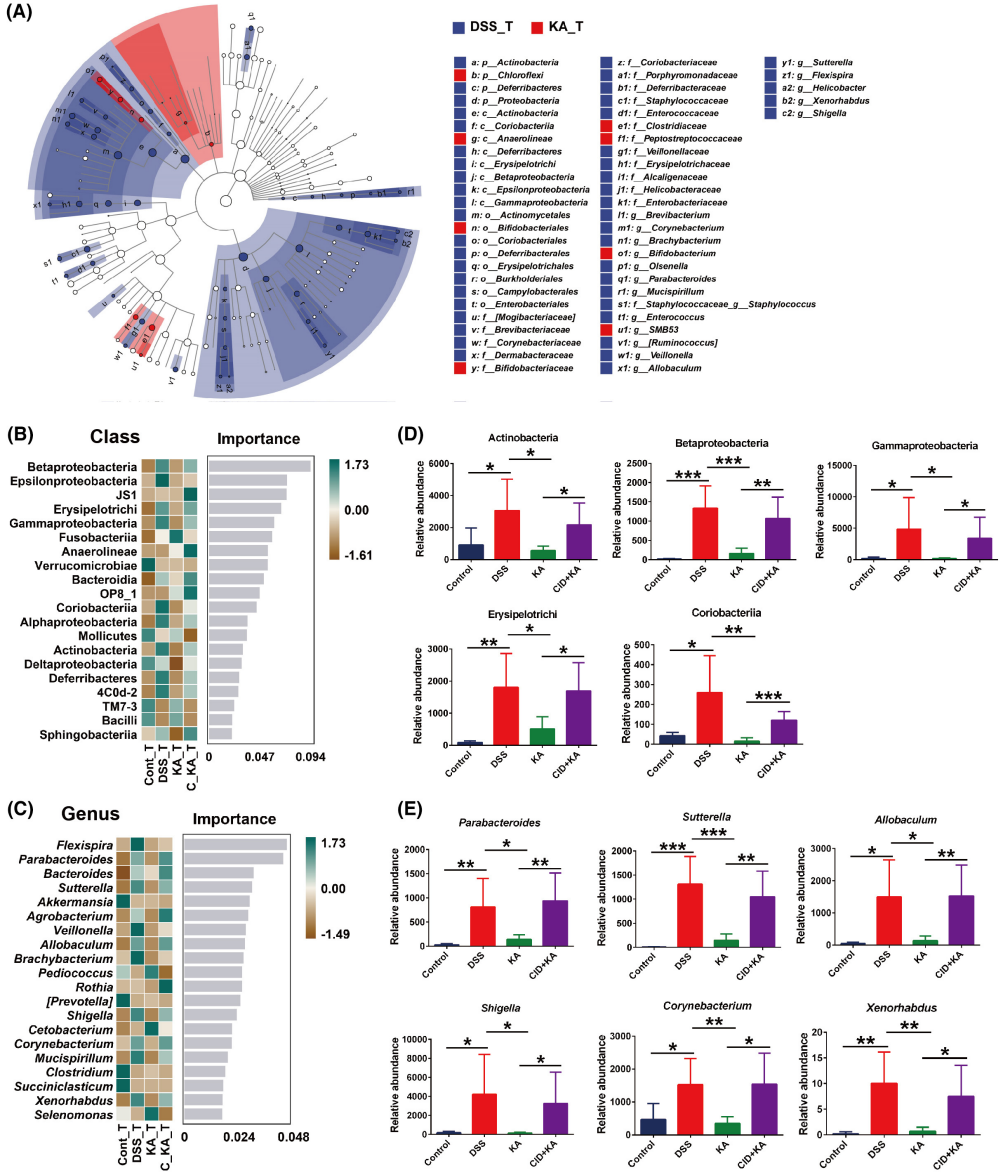
Identification of the differential species, which were associated with GPR35‐mediated KA sensing. (A) The LEfSe taxonomic cladogram indicated key bacterial alterations. The size of the circles was based on the relative abundance, the solid circle represented LDA >3.0, *P* < 0.05, and the hollow circle represented *P* > 0.05. (B, C) The importance of the gut microbiota on colitis characteristics at the class (B) and genus (C) levels was analyzed by Random Forest analysis and the top 20 taxa were displayed. (D, E) The relative abundance of the marked classes (D) and genera (E) that were closely associated with GPR35‐mediated KA sensing. The data are presented as mean ± SD. Statistical analysis was performed using the Student's *t* test. *n* = 6, *P < 0.05, **P < 0.01 and ***P < 0.001.

In order to further explore the taxonomic distribution of the marked microbiota in each experimental group, LEfSe analysis was performed. Overall, the data indicated that compared to the control group, the disorders of the multitudinous colonic microbiota were present in the model group (LDA >3.0, *P* < 0.05) (Fig. S3A). Attractively, KA significantly improved the disordered bacteria to a near‐normal state (Fig. S3B). However, this effect was dramatically blocked when host GPR35 activity was inhibited (Fig. S3C). Specifically, at the class level, Actinobacteria, Coriobacteriia, Bacteroidia, and other taxa (10 in total) were significantly enriched, whereas the abundance of TM7_3 and Verrucomicrobiae was markedly reduced in the model group compared with the control group (Fig. S3A). Among them, the dysbiosis of Actinobacteria, Beta‐/Gamma‐Proteobacteria, Erysipelotrichi, and Coriobacteriia was ameliorated by KA treatment, and this effect was markedly blocked following CID intervention (Fig. 3A and Fig. S3D). At the genus level, 16 taxa, including *Corynebacterium*, *Bacteroides*, and *Parabacteroides* were obviously enriched in the model group, and 7 taxa including *AF12*, *Anaerostipes*, and *Akkermansia* were significantly enriched in the control group (Fig. S3A). After KA treatment, the differential bacterial genera were dramatically reduced and only *Bacteroides* and *Sutterella* were enriched, while the abundance of *Akkermansia* was decreased in the KA group compared with the control group (Fig. S3B). However, CID intervention significantly reversed the effect of KA and led to the differential bacterial genus being markedly increased, including 13 taxa enriched and 9 taxa reduced in the CID + KA group (Fig. S3C).

Subsequently, Random Forest analysis was performed to further assess the specific bacterial taxa related to GPR35‐mediated KA sensing. The results showed that KA effectively ameliorated the majority of the imbalanced microflora, which was induced by DSS exposure, at the class and genus levels (Fig 3B,C). Among the top 20 most important classes for colitis characteristics, DSS lead to the abundance of Actinobacteria, Beta‐/Gamma‐Proteobacteria, Erysipelotrichi, and Coriobacteriia significantly upregulated, while this effect was markedly reversed after KA treatment (*P* < 0.05) (Fig. 3D). Additional results demonstrated that inhibition of the host GPR35 activity dramatically blocked the ameliorative effect of KA on the taxa suggested that GPR35‐mediated KA sensing was closely related to the homeostasis of the 5 classes during the progression of colitis. More importantly, in the top 20 most important genera, the obvious increase in the abundance of *Corynebacterium* (class Actinobacteria), *Allobaculum* (class Erysipelotrichi), *Parabacteroides* (class Bacteroidia), *Sutterella* (class Betaproteobacteria), *Shigella*, and *Xenorhabdus* (class Gammaproteobacteria) was shown in the model group. Consistently, KA significantly suppressed the imbalance of the 6 genera, and this effect was notably blocked by CID (*P* < 0.05) (Fig. 3E). These results demonstrated that *Corynebacterium*, *Allobaculum*, *Sutterella*, *Parabacteroides*, *Shigella*, and *Xenorhabdus* were the marked bacterial taxa that could be used to characterize UC progression and closely associated with GPR35‐mediated KA sensing.

### GPR35 plays an important role in the recovery of gut barrier integrity

**Fig. 4:**
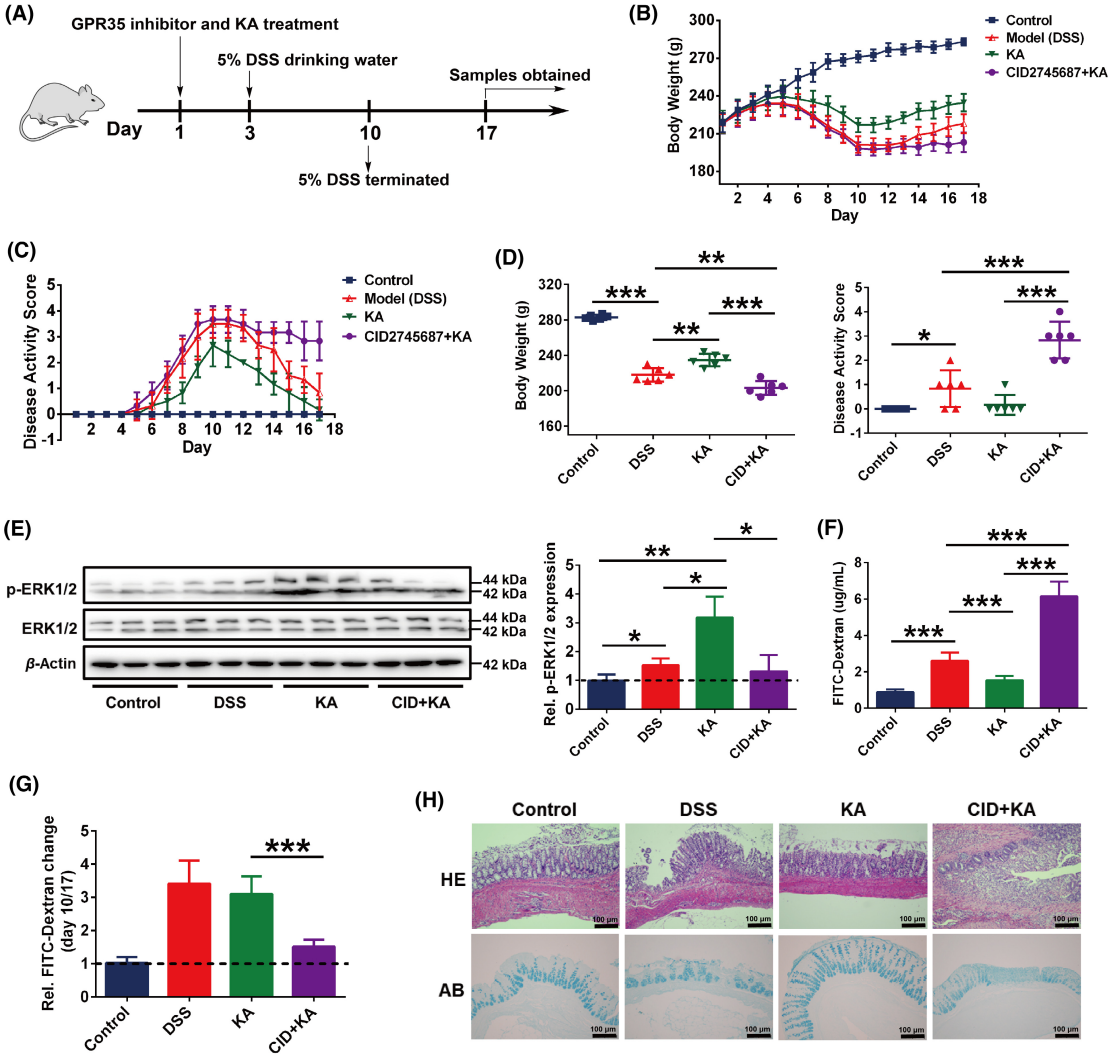
The GPR35‐mediated KA sensing is closely related to the recovery of DSS‐induced colitis. (A) Schematic representation of the experimental design used. (B, C) The body weight (B) and disease activity score (C) of each rat were monitored daily, CID represents the GPR35 specificity inhibitor CID2745687. (D) The body weight and disease activity score were analyzed on day 17. (E) Western blot and quantification analyses of the protein expression levels of ERK1/2 and p‐ERK1/2 in the rat colon tissues. (F) Intestinal permeability analysis. (G) The relative change of the intestinal permeability prior to (day 10) and following termination of DSS exposure (day 17). (H) H&E and AB staining analyses of the rat colon tissues, scale bars = 100 μm (as indicated). The data are presented as mean ± SD. Statistical analysis was performed using the Student's *t* test. *n* = 6, *P < 0.05, **P < 0.01 and ***P < 0.001.

To further confirm the important role of GPR35‐mediated KA sensing in maintaining gut microbiota homeostasis during the progression of UC, a specific administration schedule was performed as shown in Fig. 4A. Following termination of DSS administration, the mental state, exercise, and mucosanguineous feces of KA and model group rats were ameliorated daily and recovered to an almost normal state at the end of the experiment. However, the rats in the CID + KA group still exhibited a severe pathological state, although no further deterioration was observed. By assessing the daily record, it was shown that the body weights of the KA and model groups were apparently increased, and the DAS was notably reduced (Fig. 4B,C). However, no significant improvement was noted in the body weight and DAS of CID + KA group, and significant differences were noted between that group and the KA group. Moreover, the CID + KA group showed a significant decrease in body weight and an apparent increase in DAS at the end of the experiment compared with the model group (day 17, *P* < 0.05) (Fig. 4D); however, no significant differences were noted between the two groups in the determination of these parameters prior to DSS termination (day 10, *P* > 0.05) (Fig. 1D). These results demonstrated that GPR35 played an important role in the recovery of colitis.

Subsequently, the activation of GPR35 was analyzed in the colon tissues by detecting the phosphorylation level of ERK1/2. As shown in Fig. 4E, the expression of p‐ERK1/2 was significantly increased in the model group compared with the control group, which was possibly attributed to the accumulation of KA in the colon tissue during colitis progression [22]. In addition, the phosphorylation level of ERK1/2 was significantly increased in the rat colon tissue of the KA group, indicating that the activation of GPR35 was induced by KA treatment. Moreover, CID successfully inhibited KA‐induced GPR35 activation in the colon. By performing intestinal permeability analysis, it was found that the colonic permeability of the model and KA groups was dramatically alleviated to approximately 70% following DSS termination for a week compared with that noted before that period of treatment (Fig. 4F,G). Although no significant differences were noted between the model and KA groups, which was possibly attributed to the damage of the colonic barrier of the KA group being slightly lower than that of the model group prior to DSS termination (day 10) (Fig. 1F,G), KA treatment further ameliorated colonic permeability compared with that of the model group. Moreover, the inhibition of the activation of the host GPR35 dramatically intercepted the effects of KA on promoting the recovery of colonic permeability (Fig. 4F). Furthermore, histopathological examination indicated that the gut barrier integrity was obviously recovered in the model and KA groups after DSS termination, whereas the colon tissue damage, colonic crypt, and goblet cell defectiveness were markedly ameliorated (Fig. 4H). However, CID intervention led to consistent damage induced on the colonic barrier. These results demonstrated that GPR35 played an important role in the recovery of the gut barrier integrity.

### GPR35 is the key sensor in the ability of KA to maintaining gut microbiota homeostasis

**Fig. 5:**
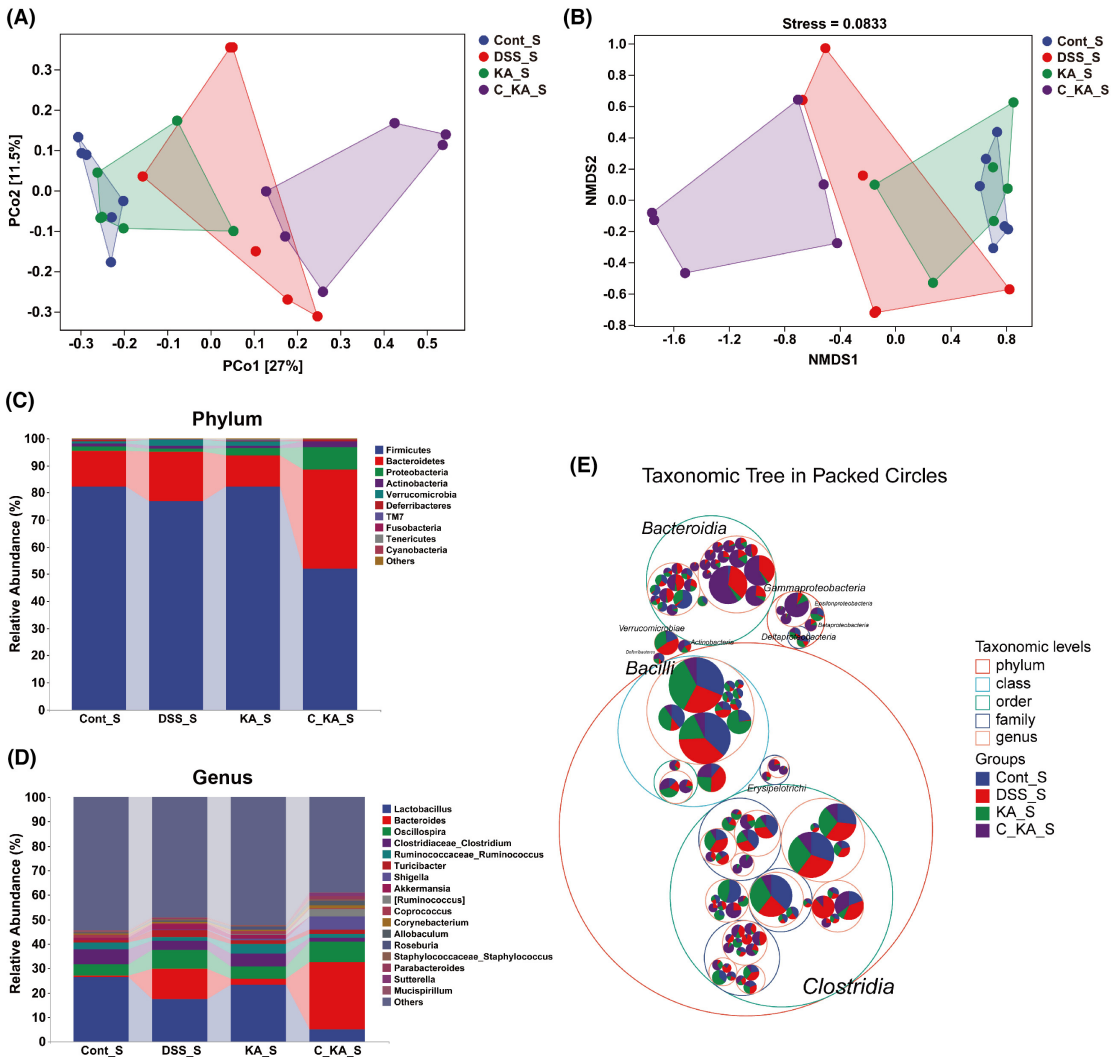
GPR35‐mediated KA sensing is closely associated with the recovery of disordered‐colonic microbiota. (A, B) The structural variation of the microbial communities across the samples was analyzed by PCoA (A) and NMDS (B). (C, D) The composition of the gut microbiota at the phylum (C) and genus (D) levels among the four groups. (E) The distribution of the taxonomic units in the classification levels was analyzed by the taxonomic tree. The suffix of “S” in each group represents that the analyzed sample was obtained on day 17.

16S rRNA gene sequencing of the colon content was performed to further confirm the importance of GPR35‐mediated KA sensing in maintaining gut microbiota homeostasis. The rarefaction curve and rank abundance curve showed that the sample size and sequencing data were acceptable, and the species in the samples exhibited a wide coverage (Fig. S4A,B). The evaluation of the α‐diversity indicated that Chao1 and Observed_species indexes exhibited significant differences among the four groups (*P* < 0.05), suggesting that the species richness was significantly altered by KA and CID during the recovery of gut microbiota homeostasis (Fig. S4C). Other indices included Shannon, Simpson, Faith_pd, Goods_coverage and Pielou_e, which indicated no significant influence on the species diversity, evenness, and coverage (*P* > 0.05). PCoA and NMDS analyses indicated that except for the CID intervention group, the KA and model groups were significantly shifted towards the control group following termination of DSS administration compared with before (Figs 2D,E and 5A,B). More importantly, no significant separation was noted between the control and KA groups, whereas the DSS and CID + KA groups exhibited obvious separation after DSS termination. Subsequent analysis of the taxonomic composition indicated no significant changes in the dominant phyla and genera prior to and following DSS termination. However, the differences noted in the bacterial spectrum among the control, model, and KA groups were notably alleviated, suggesting that the homeostasis of the colonic microbiota was recovered in the model and KA groups (Fig 5C,D). Moreover, the KA treatment group demonstrated a more complete recovery than the model group. Furthermore, the dysbacteriosis of the CID + KA group exhibited no improvement and indicated a significant change following DSS termination for a week, indicating the key role of GPR35 in maintaining gut microbiota homeostasis.

**Fig. 6:**
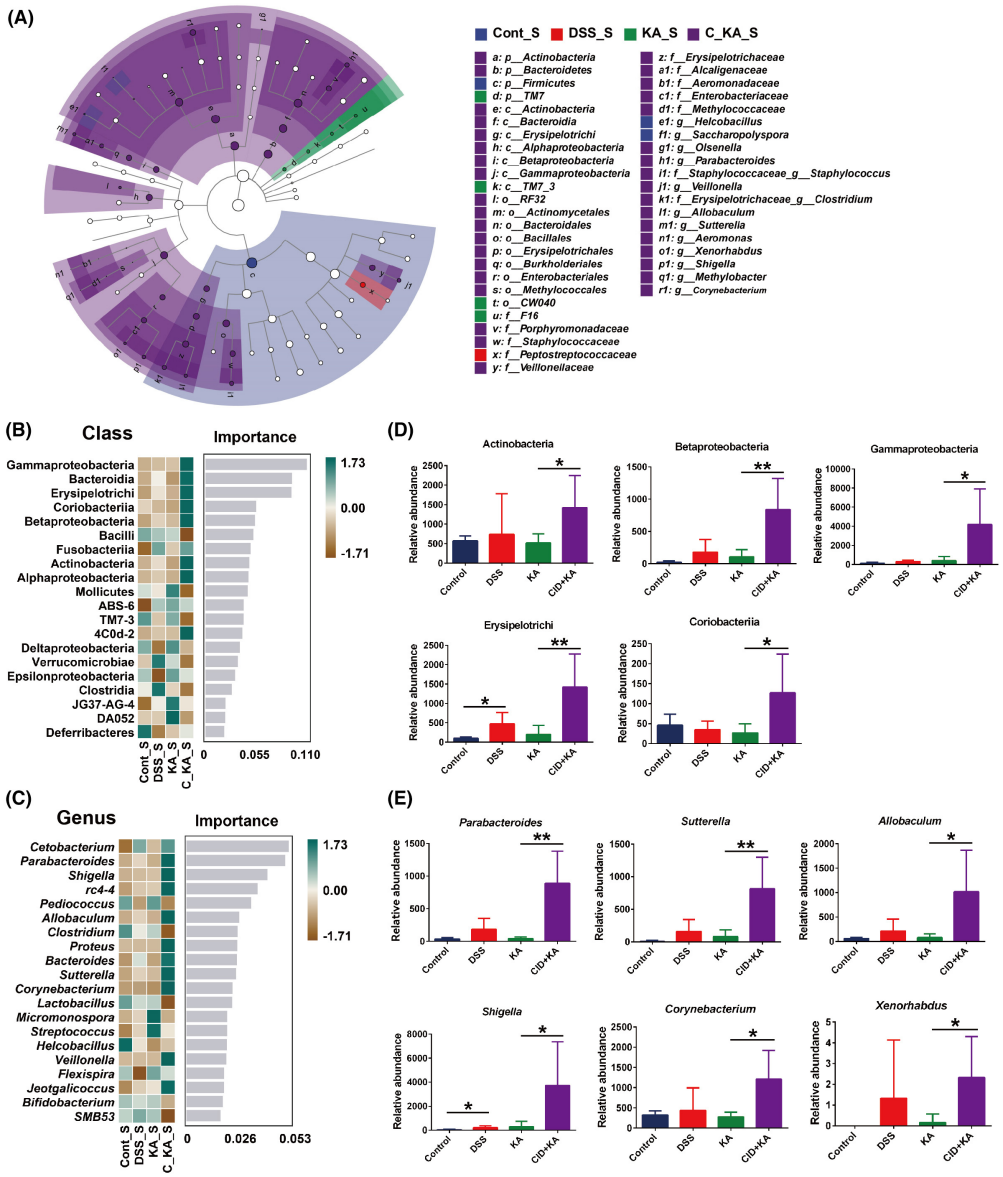
The changes of the differential species following termination of DSS exposure. (A) LEfSe analysis of the key bacterial alterations. The size of the circles is based on the relative abundance, the solid circle represents LDA >3.0, *P* < 0.05, and the hollow circle represents *P* > 0.05. (B, C) The importance of gut microbiota at the class (B) and genus (C) levels was analyzed by Random Forest analysis and the top 20 taxa were displayed. (D, E) The changes in the relative abundance of the marked classes (D) and genera (E) following termination of DSS expose. The data are presented as mean ± SD. Statistical analysis was performed using the Student's *t* test. *n* = 6, *P < 0.05 and **P < 0.01.

To further explore the taxonomic distribution of the marked microbiota after DSS termination, the taxonomic tree and LEfSe analyses were initially performed. As shown in Fig. 5E, the classes of Beta‐/Gamma‐Proteobacteria, Erysipelotrichi, Bacteroidia, and Actinobacteria were mostly enriched in the CID + KA group. However, the classes of Bacilli, Clostridia, and Verrucomicrobiae, which were widely distributed in the control, model, and KA groups, were enriched to a lesser extent in the CID intervention group. Moreover, LEfSe analysis indicated that the majority of the taxa included 6 classes and 12 genera, were significantly enriched in the CID + KA group compared to the other three groups [linear discriminant analysis (LDA) >3.0, *P* < 0.05] (Fig. 6A). These results suggested that the disorders of colonic microbiota in the GPR35‐inhibited group were consistent following termination of DSS for a week. In addition, Random Forest analysis demonstrated that the disordered profile of the colonic microbiota in the model and KA groups was not present after termination of DSS administration at the class and genus levels (Fig. 6B,C). These data also indicate that inhibiting host GPR35 activity completely blocked the recovery of colonic microbiota homeostasis induced by KA. Particularly, the classes of Actinobacteria, Beta‐/Gamma‐Proteobacteria, Erysipelotrichi, and Coriobacteriia, as well as the genera of *Corynebacterium*, *Allobaculum*, *Sutterella*, *Parabacteroides*, and *Shigella*, except for *Xenorhabdus*, were the top 20 most important taxa for the recovery of colonic homeostasis. As shown in Fig. 6D,E, despite the improvement of bacterial imbalance noted in the model group, which had earlier resulted in normal homeostasis in the KA group, the CID intervention group was not affected and exhibited a significant increase in the abundance of the taxa (*P* < 0.05). Collectively, these results further confirm that GPR35‐mediated KA sensing plays an important role in the recovery of gut microbiota homeostasis.

### GPR35‐mediated KA sensing is associated with the functional profile of the gut microbiome

**Fig. 7:**
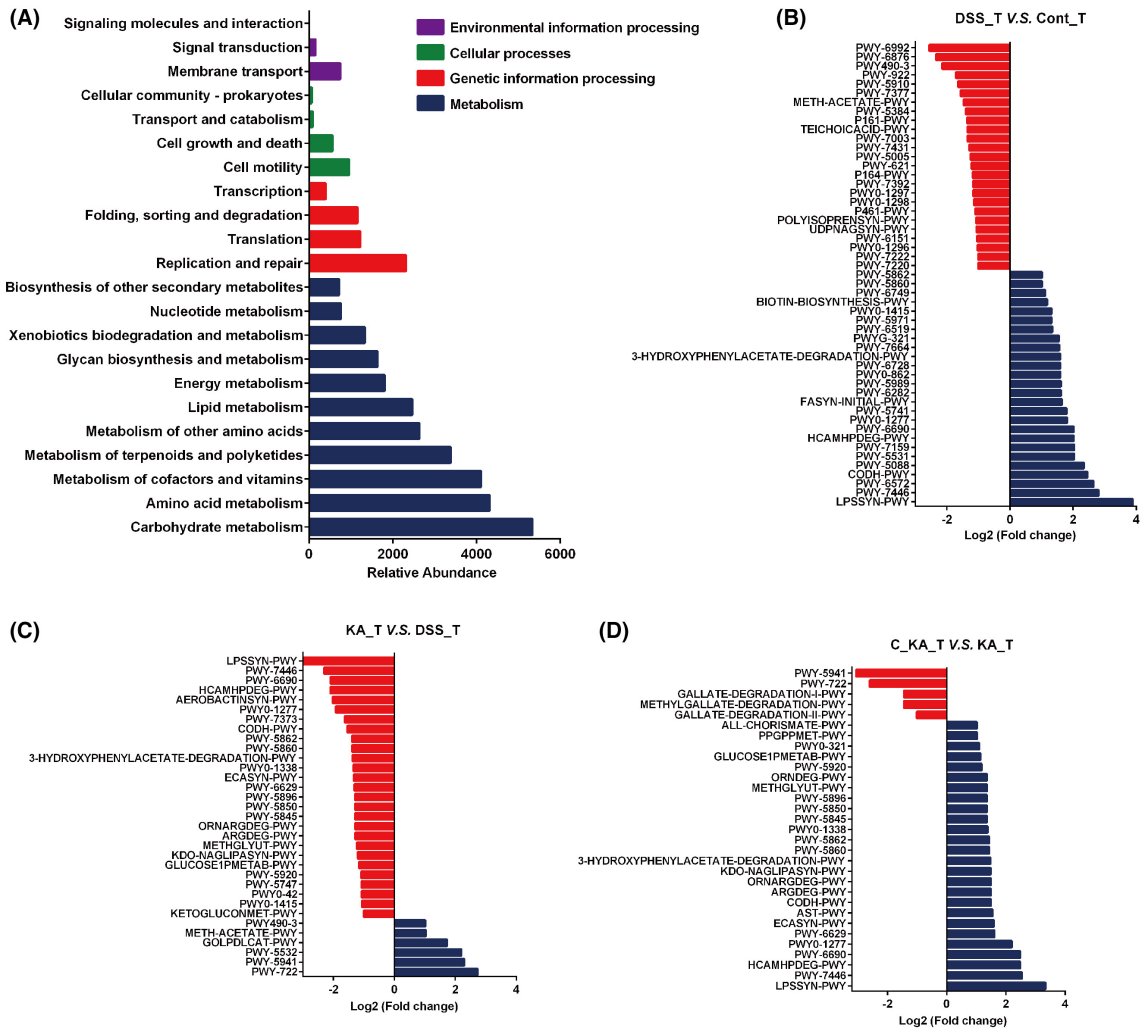
Functional and pathway enrichment analyses. (A) The functional annotation of the differential gut microbiome. (B–D) The pathway enrichment analyses were performed between the DSS_T and Cont_T groups (B), the KA_T and DSS_T groups (C), and the C_KA_T (CID2745687 + KA) and KA_T groups (D) based on the KEGG database (Fold change > 2).

To explore the functional profile of the differential gut microbiome, KEGG enrichment analysis was performed. According to the functional annotation, the gut microbiota was mainly involved in metabolic modules, including carbohydrate, amino acid, lipid, nucleotide cofactors, and vitamin metabolism, energy metabolism, glycan biosynthesis and metabolism, xenobiotic biodegradation, and metabolism (Fig. 7A). Furthermore, the differential microbiota in this study was also associated with replication and repair, translation, folding, sorting, and degradation, membrane transport, cell motility, growth, and death. Pathway enrichment analysis indicated that the superpathway of lipopolysaccharide biosynthesis (LPSSYN‐PWY), sulfoglycolysis (PWY‐7446), reductive acetyl coenzyme A pathway (CODH‐PWY), 3‐phenylpropanoate and 3‐(3‐hydroxyphenyl) propanoate degradation to 2‐oxopent‐4‐enoate (HCAMHPDEG‐PWY), cinnamate and 3‐hydroxycinnamate degradation to 2‐oxopent‐4‐enoate (PWY‐6690), 3‐phenylpropanoate and 3‐(3‐hydroxyphenyl) propanoate degradation (PWY0‐1277), 4‐hydroxyphenylacetate degradation (3‐HYDROXYPHENYLACETATE‐DEGRADATION‐PWY), superpathway of demethylmenaquinol‐6 biosynthesis I (PWY‐5860), superpathway of demethylmenaquinol‐9 biosynthesis (PWY‐5862) and superpathway of heme biosynthesis from uroporphyrinogen‐III (PWY0‐1415) were significantly upregulated, whereas methanogenesis from acetate (METH‐ACETATE‐PWY) was obviously downregulated in the model group compared with the control group (Fold change > 2, *P* < 0.05) (Fig. 7B). The changes in these pathways were notably reversed after KA treatment (Fig. 7C). However, the effects of KA on these pathways were drastically blocked when the host GPR35 activity was inhibited (Fig. 7D). These findings demonstrated that the 11 pathways were regulated by GPR35‐mediated KA sensing and were closely associated with the differential gut microbiota identified in the present study.

## Discussion

Gut barrier integrity is a key physiological variable and is maintained by the interaction of intestinal microbiota, metabolites, and their monitor, ceaselessly [5, 37]. Understanding their operating mechanism is imperative to minimize health problems and to improve the prophylaxis and therapy of UC, which remains a challenging and refractory disease in the world [38]. Our previous study demonstrated that UC caused dramatic accumulation of KA in colon tissues and discovered that GPR35 was a key guardian of KA metabolism and a core component of the defense responses against intestinal damage [22]. However, the biological effects and mechanism by which GPR35‐mediated KA sensing regulates intestinal homeostasis remain unknown. The irreplaceable role of gut microorganisms in maintaining intestinal function and integrity has been increasingly emphasized in recent years [33]. Whether GPR35‐mediated KA sensing maintains intestinal barrier integrity through regulating the homeostasis of microbiota. However, appropriate evidence to support this view is unavailable in the literature.

Therefore, in the present study, we established a DSS‐induced rat colitis model and a GPR35‐specific inhibitor (CID) was used. We found that DSS stimulation resulted in rat's bodyweight loss, DAS increase and intestinal barrier destruction in all groups. However, importantly, KA administration significantly alleviated these pathological changes compared to model group. Meanwhile, inhibiting the activation of colon GPR35 markedly blocked the effects of KA on colitis progression. Furthermore, following termination of DSS exposure, the GPR35 inhibition group showed no effects, despite the alleviation of the defective gut barrier in the KA and model groups. This suggests the importance of GPR35‐mediated KA sensing in maintaining intestinal homeostasis. Subsequent 16S rRNA analysis indicated that although the α‐diversity of the gut microbiota was not obviously influenced after KA and CID treatment, the profiles of the bacterial taxa exhibited significant differences. Notably, through PCoA and NMDS analyses we found that the significant separation of gut microbiota between the control and model groups was dramatically reversed by KA treatment, while this effect was completely blocked following inhibition of the host GPR35 activity. This evidence supports a causal relationship between the gut microbiome and GPR35‐mediated KA sensing. Indeed, subsequent studies demonstrated that KA significantly ameliorated the disorder of the colonic microbiota, which was markedly blocked following inhibition of host GPR35. No obvious improvement in dysbiosis was noted in the GPR35 inhibition group despite the termination of the DSS exposure. These findings further confirmed the close association between GPR35‐mediated KA sensing and gut microbiota homeostasis.

More importantly, using LEfSe and Random Forest analyses, we identified 5 bacterial classes, including Actinobacteria, Beta‐/Gamma‐Proteobacteria, Erysipelotrichi, and Coriobacteriia, and 6 genera, including *Corynebacterium*, *Allobaculum*, *Parabacteroides*, *Sutterella*, *Shigella*, and *Xenorhabdus*, which were the marked bacterial taxa characterizing the progression and outcome of UC and regulated by GPR35‐mediated KA sensing. Actinobacteria is one of the four major phyla of gut microbiota. Even though it represents only a small percentage of the gut microorganisms, its pivotal role in gut homeostasis and gastrointestinal diseases has attracted considerable attention [39]. At present, the genus *Bifidobacterium* in the class Actinobacteria has received the most universal favoritism for its beneficial effects in various pathological conditions [40]. In this study, we also observed a significant reduction in the abundance of *Bifidobacterium* following the development of colitis (Fig. S3A), which was markedly remitted after KA treatment (Fig. 3A). However, there was no significant difference between the KA and CID + KA groups regarding the abundance of *Bifidobacterium*. This implies that the improvement of *Bifidobacterium* in the KA group was more likely a consequence of the recovery of intestinal homeostasis, while no definite causal correlation was present with the GPR35‐mediated KA sensing process. Similarly, KA significantly suppressed the increase of *Corynebacterium* induced by DSS, and this effect was dramatically blocked when the host GPR35 activity was inhibited. Moreover, following termination of the DSS exposure, the reduction in the abundance of *Corynebacterium* in the KA and model groups was not observed in the GPR35 inhibition group, suggesting a close relationship between *Corynebacterium* and GPR35‐mediated KA sensing. As a genus of Actinobacteria, *Corynebacterium* is generally considered an important primary and opportunistic pathogen [41]. Currently, the role of *Corynebacterium* in the occurrence and development of UC is still under debate. However, *Corynebacterium* has been shown to be associated with primary sclerosing cholangitis and IBD, while it has also been proposed to be involved in the pathogenesis of chronic enteropathies [42].

The results of the present study indicated that *Sutterella*, *Shigella*, and *Xenorhabdus* were the key genera that resulted in the significant association of the Beta‐/Gamma‐Proteobacteria with GPR35‐mediated KA sensing. *Sutterella* was initially disregarded as a commensal bacterium due to its ability to induce a mild or negligible inflammatory response and not alter epithelial monolayer integrity. However, recent epidemiological studies demonstrated that mice could pass on their susceptibility intestinal diseases, including IBD, to their offspring through the *Sutterella* species [43]. Moon *et al*. indicated that *Sutterella* caused an IgA‐defective condition in intestinal mucosa through the secretion of IgA protease. IgA deficiency is considered the crucial pathogen responsible for the development of various intestinal diseases, including chronic diarrhea, Crohn's disease, and UC [44]. Therefore, compared with directly inducing inflammation, *Sutterella* is more likely to impair the functionality of the intestinal antibacterial immune response, notably due to its capacity to limit intracellular bacterial species [45]. This suggests that GPR35‐mediated KA sensing can possibly provide a conducive environment for the recovery of gut homeostasis by inhibiting the immune‐impairing species of *Sutterella*. At present, *Shigella* is considered the culprit causing bacillary dysentery, while its abundance with a significant increase has also been observed in UC patients [46]. In contrast to *Sutterella*, the main pathogenic factors of *Shigella* are its invasiveness and endotoxin, which increase the permeability of the intestinal mucosa [47]. *Shigella* can weaken the sealing of the epithelial cell layer and aggravate the inflammatory response by changing the composition of tight junction‐associated proteins, ultimately leading to diarrhea with mucus pus and blood [48]. These findings support the hypothesis that *Shigella* is also one of the major contributors to the destruction of the gut barrier integrity during the development of UC. In the present study, the powerful inhibitory effect of KA on *Shigella* demonstrate that GPR35‐mediated KA sensing contribute to the resistance against the invasion of the pathogenic microorganisms that can directly damage the intestinal mucosa. Compared to the other 5 marked genera, *Xenorhabdus* seemed trivial for its abundance and was almost undetectable in the healthy rats. Currently, limited evidence has reported that links between *Xenorhabdus* and UC pathogenesis, although its pathogenicity in the host has been previously proposed [49].

The mucous layer is an important component of the intestinal barrier that can resist the invasion of pathogenic microorganisms, and its importance is self‐evident [50]. A previous study indicated that *Allobaculum* (class Erysipelotrichi) secretes a wide repertoire of mucin O‐glycan targeting carbohydrate active enzymes (CAZymes), which allow it to effectively degrade and feed on intestinal mucins [51]. The uncontrolled proliferation of *Allobaculum* can destroy the host mucus layer. In this study, we observed that the abundance of *Allobaculum* was dramatically increased, and the colonic barrier was damaged following the development of colitis. Although the reasons for intestinal barrier destruction are complex, the massive degradation of the mucus layer by *Allobaculum* is undoubtedly one of the main contributors to this effect. This further enhanced the significance of GPR35‐mediated KA sensing in maintaining gut homeostasis for the regulation of *Allobaculum*. In addition, the significant increase in the abundance of *Parabacteroides* (class Bacteroidia) in the murine model of DSS‐induced colitis has also been reported [52]. It is noteworthy that current views on the role of *Parabacteroides* in the pathogenesis of UC are contradictory. Some studies have shown that *Parabacteroides* play an anti‐inflammatory role in the intestinal microbiome, which is beneficial to the development of UC in mice [53]. However, accumulated evidence demonstrates that under specific conditions, *Parabacteroides* can promote intestinal inflammation in mice rather than alleviate it. In addition, gavage with *Parabacteroides* aggravated DSS‐induced colitis and increased the susceptibility of the mice to IBD [54]. Although the potential contribution of *Parabacteroides* to the pathogenesis of UC remains unclear, investigation of this condition in animal models has highlighted its severity. The regulation of GPR35‐mediated KA sensing on *Parabacteroides* may possibly benefit the recovery of gut homeostasis.

Additionally, functional profile analysis revealed that the differential gut microbiome was involved in multiple biological functions, notably carbohydrate, amino acid, cofactor, and vitamin metabolism. The maintenance of GPR35‐mediated KA sensing on gut microbiota homeostasis undoubtedly exerts a positive effect on the aberrant functions of UC. Moreover, we also found that GPR35‐mediated KA sensing was closely associated with 11 signaling pathways via pathway enrichment analysis based on the differential gut microbiome. This implied that these pathways may be the key link between the gut microbiota and the integrity of the colonic barrier maintained by GPR35‐mediated KA sensing during UC. However, further experimental verification is necessary.

## Conclusion

In this study, we demonstrated that GPR35‐mediated KA sensing was a necessary component in maintaining gut barrier integrity against DSS‐induced gut damage. This process partly regulates gut microbiota homeostasis. Subsequent studies indicated that the disordered‐colonic microbiome was effectively reversed by KA, and this effect was completely blocked following inhibition of host GPR35 activity. More importantly, 5 classes and 6 genera were identified as the marked bacterial taxa characterizing the progression and outcome of UC and were closely associated with GPR35‐mediated KA sensing. These findings highlight that GPR35‐mediated KA sensing is an essential defender against gut microbiota disorder.

## Conflict of interest

The authors declare no conflict of interest..

### Peer review

The peer review history for this article is available at https://www.webofscience.com/api/gateway/wos/peer‐review/10.1002/2211‐5463.13673.

## Author contributions

DW designed and performed experiments, analyzed and interpreted data, and wrote the manuscript. WW assisted in 16S rRNA sequencing data analysis. XB and CX assisted in performing animal experiments. JQ and JS assisted in performing histopathological examination. JH, JL and PL commented and proofread the manuscript. BX commented the manuscript, administrative support and study supervision. All authors contributed to the article and approved the submitted version.

## Supporting information


**Fig. S1.** Schematic representation of rat disposal process in each group.
**Fig. S2.** α‐diversity analysis of gut microbiota by Observed_species, Shannon, Simpson, Faith_pd, Goods_coverage and Pielou_e indexes.
**Fig. S3.** The LEfSe analysis of the differential gut microbiome in Cont_T and DSS_T groups (A), Cont_T and KA_T groups (B), Cont_T and C_KA_T groups (C), KA_T and C_KA_T groups (D). The size of the circles was based on the relative abundance, the solid circle represented LDA >3.0, *P* < 0.05, and the hollow circle represented *P* > 0.05.
**Fig. S4.** (A, B) Rarefaction curve (A) and Rank abundance curve (B) of each group. (C) α‐diversity analysis of gut microbiota by Chao1, Observed_species, Shannon, Simpson, Faith_pd, Goods_coverage and Pielou_e indexes.

## Data Availability

The original contributions presented in the study are included in the article/Supplementary Material. Further inquiries can be directed to the corresponding authors.
