# Supplementary material for: GPR35‐mediated kynurenic acid sensing contributes to maintenance of gut microbiota homeostasis in ulcerative colitis
Source: FEBS Open Bio. 2023 Jul 14;13(8):1415–33. doi: 10.1002/2211-5463.13673 (PMC10392069; doi:10.1002/2211-5463.13673)

Supplementary Material

GPR35-mediated kynurenic acid sensing contributes to maintenance of gut microbiota homeostasis in ulcerative colitis

Di Wang[1,2, †], Wenbao Wang[4, †], Xue Bing[2], Chenguang Xu[2], Jiahua Qiu[2], Jiangang Shen[2], Jinwen Huang[2], Junda Li[2], Pi Liu[2], Biao Xie[2,3]*

[1] School of Life and Health Technology, Dongguan University of Technology, Dongguan 523808, P.R. China

[2] Department of Gastroenterology, Affiliated Longhua People's Hospital, Southern Medical University（People's Hospital of Longhua）, Shenzhen 518109, P.R. China

[3] Department of Gastroenterology, Guangzhou Eighth People’s Hospital, Guangzhou Medical University, Guangzhou 510440, P.R. China

[4] College of Pharmacy, Qiqihar Medical University, Qiqihar 161006, P.R. China

*Correspondence should be addressed to Biao Xie, Chief physician,
Department of Gastroenterology, People's Hospital of Longhua, NO.38 Jinglong Construction Road, Longhua District, Shenzhen 518109, China. Tel/Fax: +0755-27741585; E-mail: xiebiaohxq@163.com

[†] These authors contributed equally to this work

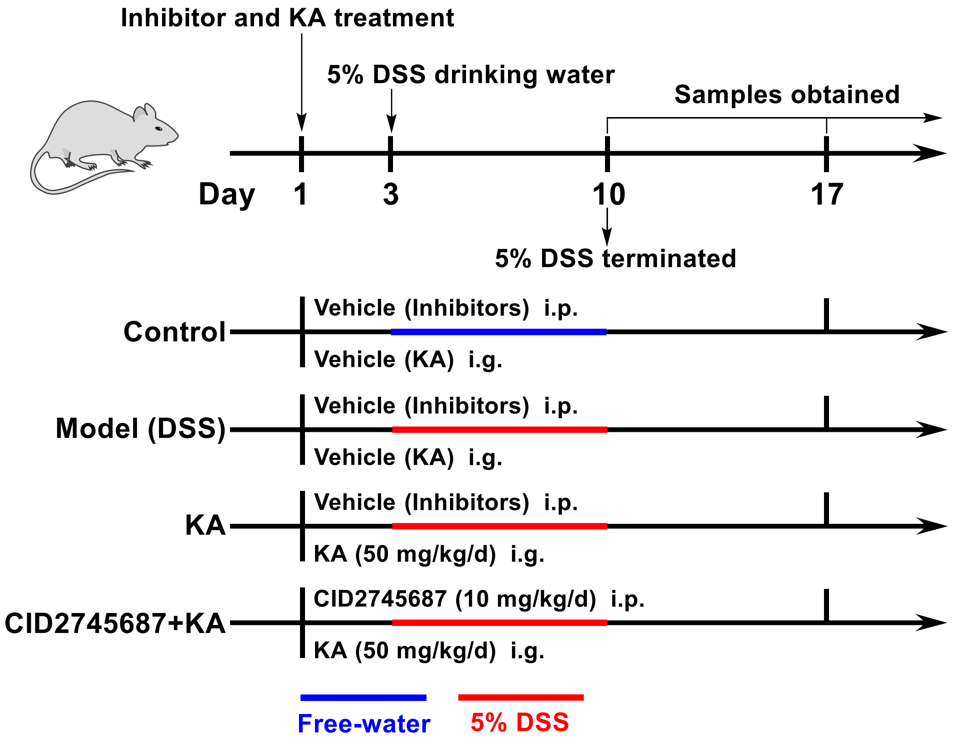

Figure S1. Schematic representation of rat disposal process in each group.

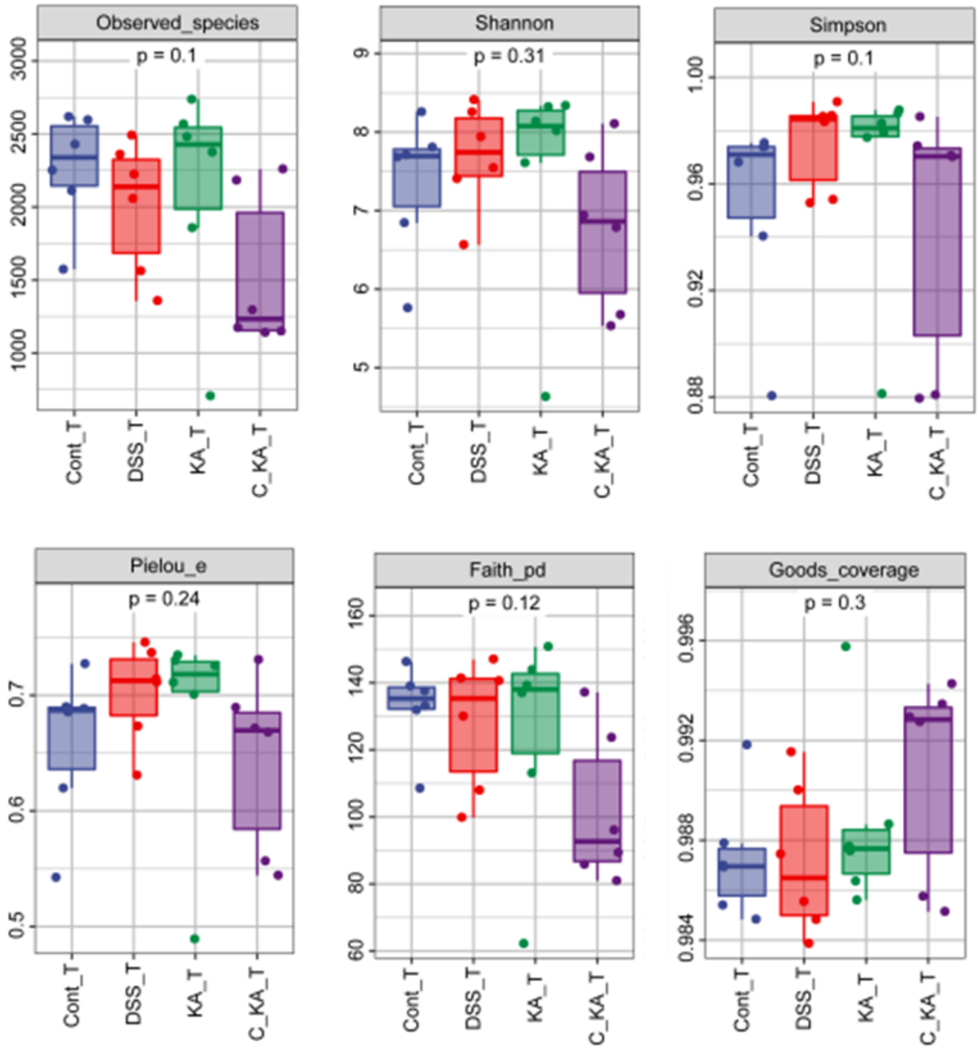

Figure S2. α-diversity analysis of gut microbiota by Observed_species, Shannon, Simpson, Faith_pd, Goods_coverage and Pielou_e indexes.

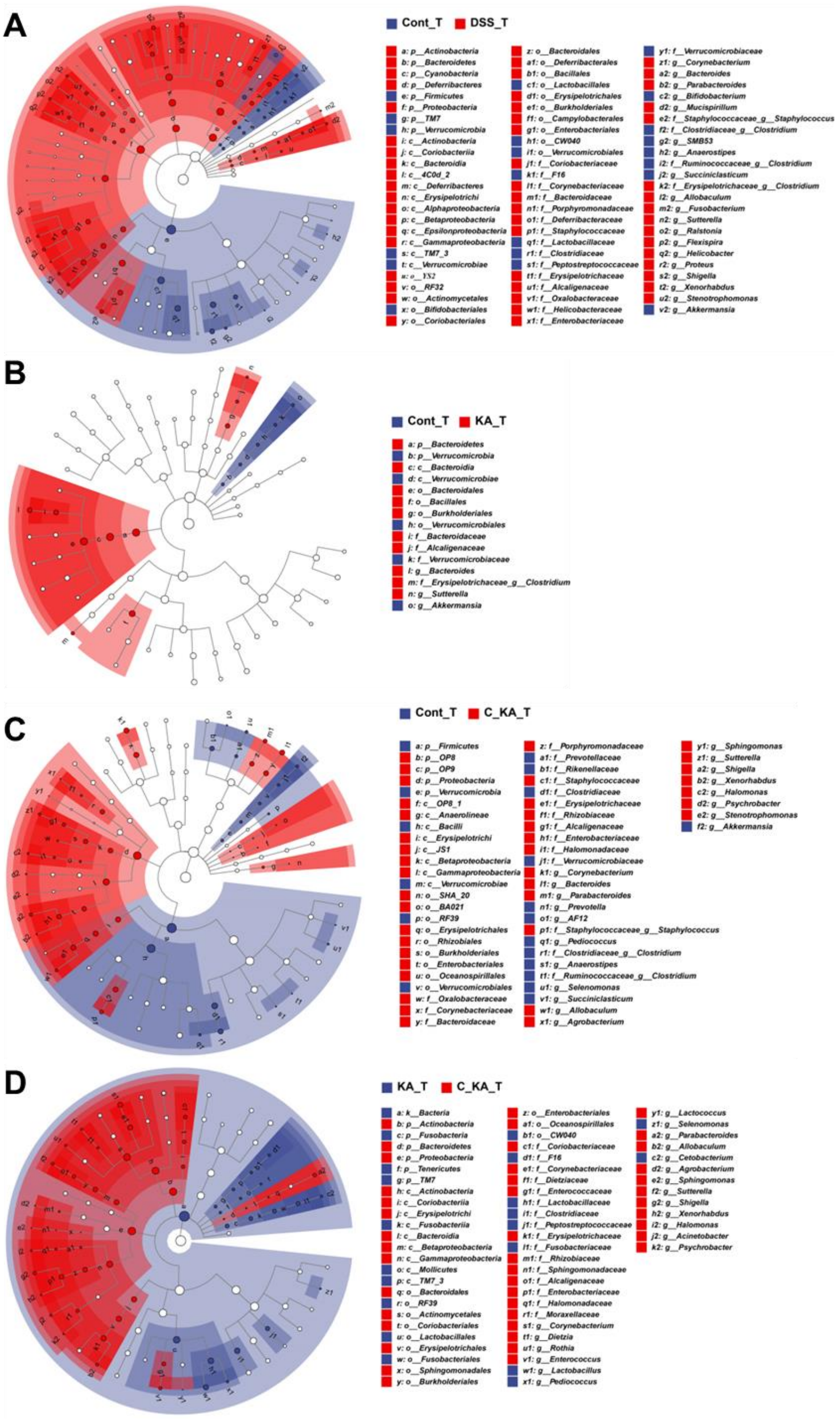

Figure S3. The LEfSe analysis of the differential gut microbiome in Cont_T and DSS_T groups (A), Cont_T and KA_T groups (B), Cont_T and C_KA_T groups (C), KA_T and C_KA_T groups (D). The size of the circles was based on the relative abundance, the solid circle represented LDA > 3.0, $p < 0.05$, and the hollow circle represented $p > 0.05$.

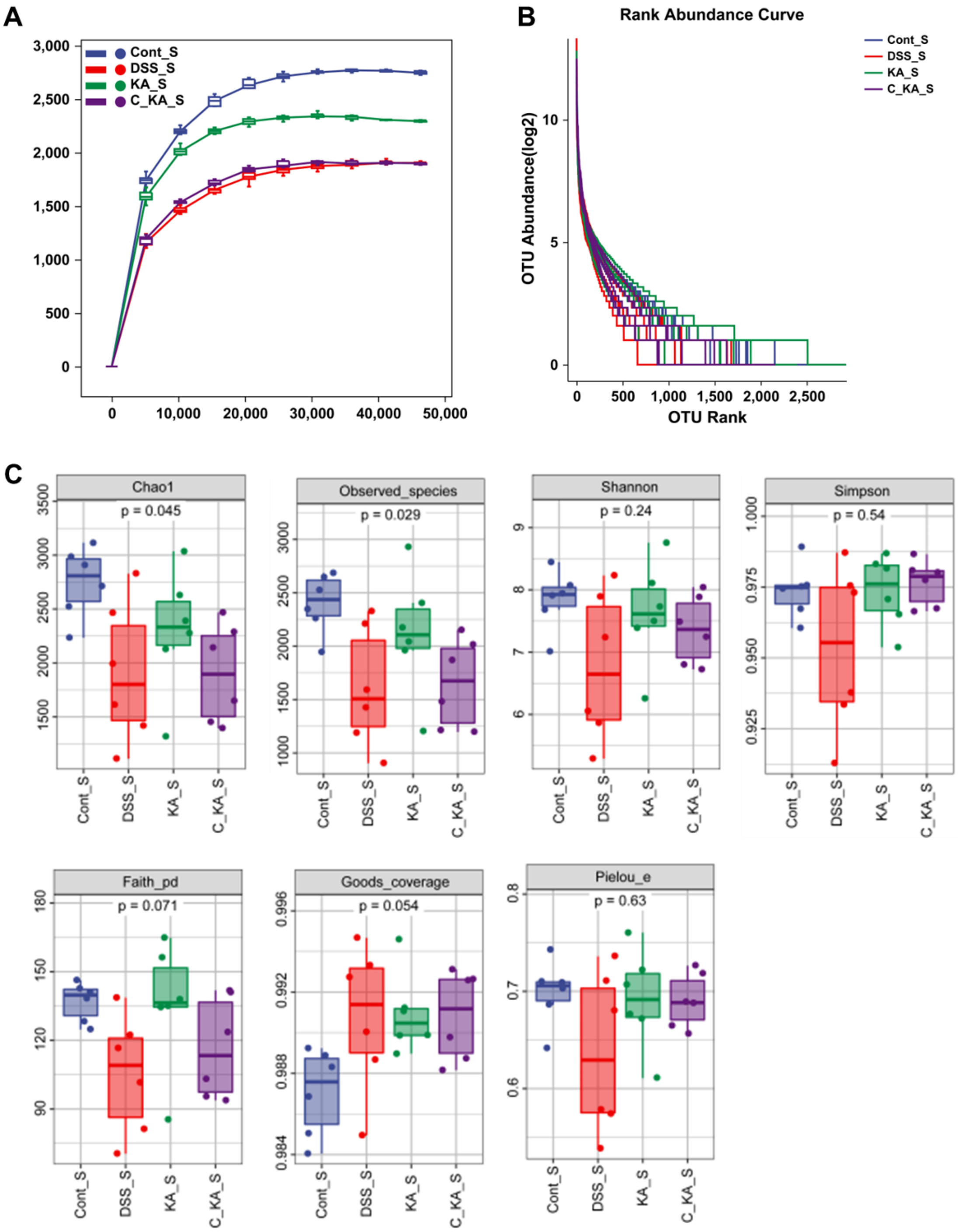

Figure S4. (A, B) Rarefaction curve (A) and Rank abundance curve (B) of each group. (C) α-diversity analysis of gut microbiota by Chao1, Observed_species, Shannon, Simpson, Faith_pd, Goods_coverage and Pielou_e indexes.

The raw unedited western blot images presented in Figure 1E

p-ERK1/2 ERK1/2 *β*-actin

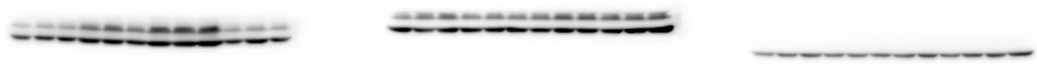

The raw unedited western blot images presented in Figure 4E

p-ERK1/2 ERK1/2 *β*-actin

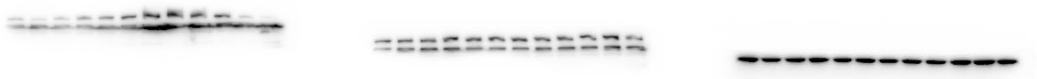

Supplement: Supplementary file 1 — Fig. S1. Schematic representation of rat disposal process in each group. Fig. S2. α‐diversity analysis of gut microbiota by Observed_species, Shannon, Simpson, Faith_pd, Goods_coverage and Pielou_e indexes. Fig. S3. The LEfSe analysis of the differential gut microbiome in Cont_T and DSS_T groups (A), Cont_T and KA_T groups (B), Cont_T and C_KA_T groups (C), KA_T and C_KA_T groups (D). The size of the circles was based on the relative abundance, the solid circle represented LDA >3.0, P < 0.05, and the hollow circle represented P > 0.05. Fig. S4. (A, B) Rarefaction curve (A) and Rank abundance curve (B) of each group. (C) α‐diversity analysis of gut microbiota by Chao1, Observed_species, Shannon, Simpson, Faith_pd, Goods_coverage and Pielou_e indexes. [file FEB4-13-1415-s001.pdf]
